# Supplementary material for: Mitigation of salinity stress in yarrow (Achillea millefolium L.) plants through spermidine application
Source: PLoS One. 2024 Jun 26;19(6):e0304831. doi: 10.1371/journal.pone.0304831 (PMC11206933; doi:10.1371/journal.pone.0304831)
Supplement: Attachment — Submitted filename: Reviewer Comments.docx [file pone.0304831.s002.docx]

**Dear Dr. Gururani,**

I am writing to express my heartfelt gratitude for your invaluable assistance and guidance in handling our manuscript. I would like to extend my sincere appreciation to the anonymous reviewers who dedicated their time and expertise to evaluate our manuscript. I am also grateful for the reviewers thoroughness in carefully reading the manuscript and providing detailed feedback, which has significantly improved the final version of the paper.

Here is a point-by-point response to the reviewer comments

**Reviewer 1#**

(1) Increase the depth of data analysis in the abstract and results sections

- Thank you for your constructive feedback which has helped improve our manuscript.
- We have revised the abstract to provide more detailed analysis of our key results, including response patterns of different parameters to salinity and spermidine treatments and their interactions. Quantitative values are now included to better demonstrate treatment effects.
- For each measured parameter in the results section, we have supplemented the text with the corresponding results. Additionally, we have analyzed response trends across treatments and added sentences describing significant findings from the data. This aims to provide a more comprehensive presentation and interpretation of our results as per your suggestion.

(2) The description of index testing in materials and methods is too detailed.

- Based on your comment, we have revised the descriptions of all measurement methods, we removed unnecessary details. The revised descriptions focus only on the key steps and calculations performed.

(3) Superscript of plus or minus signs in line 359

- They were revised.

(4) The figure lacks the abscissa title, and the error bar is supplemented.

- Abscissa title and error bars were added in the figures.

(5) I don't understand the meaning of Table 2.

- You raise a valid point - the ANOVA table on its own does not fully convey the treatment effects or aid in understanding the broader findings and conclusions of the study. Therefore, we have decided to remove Table 2 from the manuscript as per your suggestion.

**Reviewer #2:**

1. The authors mentioned "spermidine hormone" many times in the manuscript. However, spermidine is not a hormone. It should be called "spermidine" or "spermidine treatment." Please correct them throughout the manuscript.

- Thank you for your constructive feedback which has helped improve our manuscript. The hormone word was removed.

2. The authors' findings outlined the impact of spermidine and salinity on various parameters such as electrolyte leakage, chlorophyll levels, and antioxidant enzyme activities. However, the inclusion of phenotype photographs is absent. Please include photographs presenting the differences between plants under normal conditions, those exposed solely to salt stress, and those treated with salt and spermidine.
Regarding the figures:

- The following Figure was added in the manuscript.

**Fig. 1.** Effect of spermidine and salinity on plant growth in *Achillea millefolium*. (a) Control (0 mM NaCl, 0 μM spermidine); (b) 3 μM spermidine with 0 mM NaCl; (C) 3 μM spermidine with 90 mM NaCl; and (d) 90 mM NaCl with 0 μM spermidine.

1. Add error bars to indicate data variability. (Figures 1 to 5)

- The error bars were added to Figs 1-5.

2. Use color-blind-friendly palettes like blue and orange to improve accessibility. (Figures 1 to 5)

- The color of the figs. Were updated.

3. Add significant symbols to the figures 6 and 7.

- The significant letters were added in these two figures.

4. Describe the significant letters' meaning in the figure legend—an example here: Figure 1 Interaction effects of spermidine and salinity on proline content of yarrow. Different letters indicate xxx, and bars show the xxx.

- It was added as “Bars represent the standard error, and different letters above bars indicate significance based LSD test (p<0.01).”

Others:

1. Keep the formats of subtitles consistent (Line 268). Decide whether subtitles will end with a period or not.

- The formats of subtitles were edited to be consistent.

2. All the subtitles in the results part need to be changed. The title should describe the significant findings of each section. Here is an example: (Line 231) "Electrolyte leakage" can be changed to "Spermidine Reduces Electrolyte Leakage Under Salinity Stress." Please correct them throughout the whole manuscript.

- The subtitles were revised based on your suggestion.

Thanks again,

Corresponding author,
